# Does endodontics influence radiological detection of external root resorption? an in vitro study

**DOI:** 10.1186/s12903-023-02871-w

**Published:** 2023-04-17

**Authors:** C. Parrales-Bravo, S. P. Friedrichsdorf, C. Costa, J. B. Paiva, A. Iglesias-Linares

**Affiliations:** 1 School of Dentistry, University of Guayaquil, Guayaquil, Ecuador; 2 School of Dentistry, Complutense, BIOCRAN Research Group, Complutense University of Madrid, Madrid, Spain; 3 School of Dentistry, University of São Paulo, São Paulo, Brazil

**Keywords:** Cone-beam computed tomography, Digital radiography, Endodontic teeth, Non-endodontic teeth, Root resorption

## Abstract

**Background:**

External root resorption (ERR) has a multifactorial etiology and is difficult to diagnose, which means that is continues to be of research interest. This work mainly aims to determine whether external root resorption can be differentially detected in root-filled versus non-endodontically treated teeth using digital periapical radiography (DPR) and cone-beam computed tomography (CBCT).

**Methods:**

The Checklist for Reporting In-vitro Studies (CRIS) guidelines were followed throughout this study. This experiment highlights the preparation and generation of standardized synthetic teeth measured on three-dimensional records converted into Digital Imaging and Communication on Medicine (DICOM) file format. Twelve replicate maxillary incisors were randomized into two groups: (G1) six non-endodontically treated, and (G2) six endodontically treated teeth. In both groups, actual tooth lengths of all specimens were measured and compared with measurements obtained using DPR and CBCT. Simulated ERR lesions [0.12, 0.18, 0.20 mm × 0.5 mm depth in the mesial, distal and palatal apical regions] were created progressively, radiographic images were recorded, and 24 DPRs and 96 CBCTs were obtained in total. Eight blinded, previously calibrated researchers made a total of 1920 measurements (using Horos Software). Data were analyzed using the Shapiro–Wilk, ANOVA, Kruskal–Wallis and Wilcoxon rank post-hoc tests [Bonferroni correction in multiple comparison tests (*p* < 0.05)].

**Results:**

ICC values for intra- and inter-examiner agreement were appropriate. DPR overestimated ERR detection compared to the actual and CBCT measurements [Mean diff = 0.765 and 0.768, respectively]. CBCT diagnosis of ERR lesions in specimens without root canal treatment was significantly more accurate than DPR diagnoses on both non-endodontically and endodontically-treated specimens [*p* = 0.044; *p* = 0.037, respectively]. There was an 18.5% reduction in sensitivity in all DPR diagnoses made on endodontic teeth versus those made on non-endodontically treated teeth. For the smallest ERR lesions, this sensitivity was even more marked, with 27.8 and 25% less sensitivity, respectively.

**Conclusions:**

The results of this study highlight that both CBCT and DPR are good diagnostic methods for ERR. Nevertheless, root canal filling material influences diagnostic capability in ERR. The clinical significance was that the presence of intracanal material reduces the detection and diagnosis of ERR by DPR in teeth with root canal treatment.

**Supplementary Information:**

The online version contains supplementary material available at 10.1186/s12903-023-02871-w.

## Background

External root resorption (ERR) has a multifactorial etiology and is difficult to diagnose, which means that is continues to be of research interest. The ERR is defined as the permanent loss of dental tissue, affects the cementum and dentine, and in the most severe forms, it may even compromise the dental pulp tissue [1, 2]. A growing body of research suggests that the upper anterior teeth are those most susceptible to root resorption in the apical third (ERR) secondary to the mechanical stimuli generated by orthodontic forces [2–6]. However, a number of other different predisposing factors have been associated with the triggering of this pathological phenomenon [7]; more particularly, the influence of specific biological or genetic factors, together with certain treatment-dependent factors [8], has been associated with this iatrogenic effect in the context of orthodontic treatment [9]. With regard to the latter factors, it has been shown that the effect of orthodontic movement on vital or root-filled teeth varies according to their susceptibility to ERR [10]. Other evidence however shows that endodontic teeth are more prone to be affected by ERR [2], while other authors have pointed out that teeth lacking pulp tissue have a protective effect against ERR when subjected to orthodontic movement [3, 11–13]. Some explanatory hypotheses have been suggested to explain these data, although there is still no conclusive information in the literature [6].

In this context, one of the most common difficulties in the detection of ERR is the visualization and accuracy of radiographic images [14, 15]. This pathology is difficult to diagnose due to scatter and beam-hardening artifacts caused by surrounding high-density structures, as well as the use of metal posts, tooth restorations and root filling materials [16]. High bone density in the posterior region of the jaw, which prevents visualization of minor changes in root shortening, should also be considered [17]. Other factors that affect radiographic detection of ERR are the density and contrast of the images, the dental position, and the shape of the lesion in three dimensions [18]. Finally, tooth angulations and root torsion [19] and the presence and density of gutta-percha in the root canal can also make it difficult to detect external root surface lesions radiographically [20]. Overall, there is very little in vitro research evaluating the influence of root filling material on the diagnosis of ERR by radiological imaging. Therefore, the null-hypothesis was that the root-filling does not influence the ERR detection. The aim of the present study was to assess, using two different radiographic methods, whether differential perceptions in diagnosis of ERR on vital / endodontic teeth are a potential source of misdiagnosis.

## Methods

### Study design and specimens

**Fig. 1:**
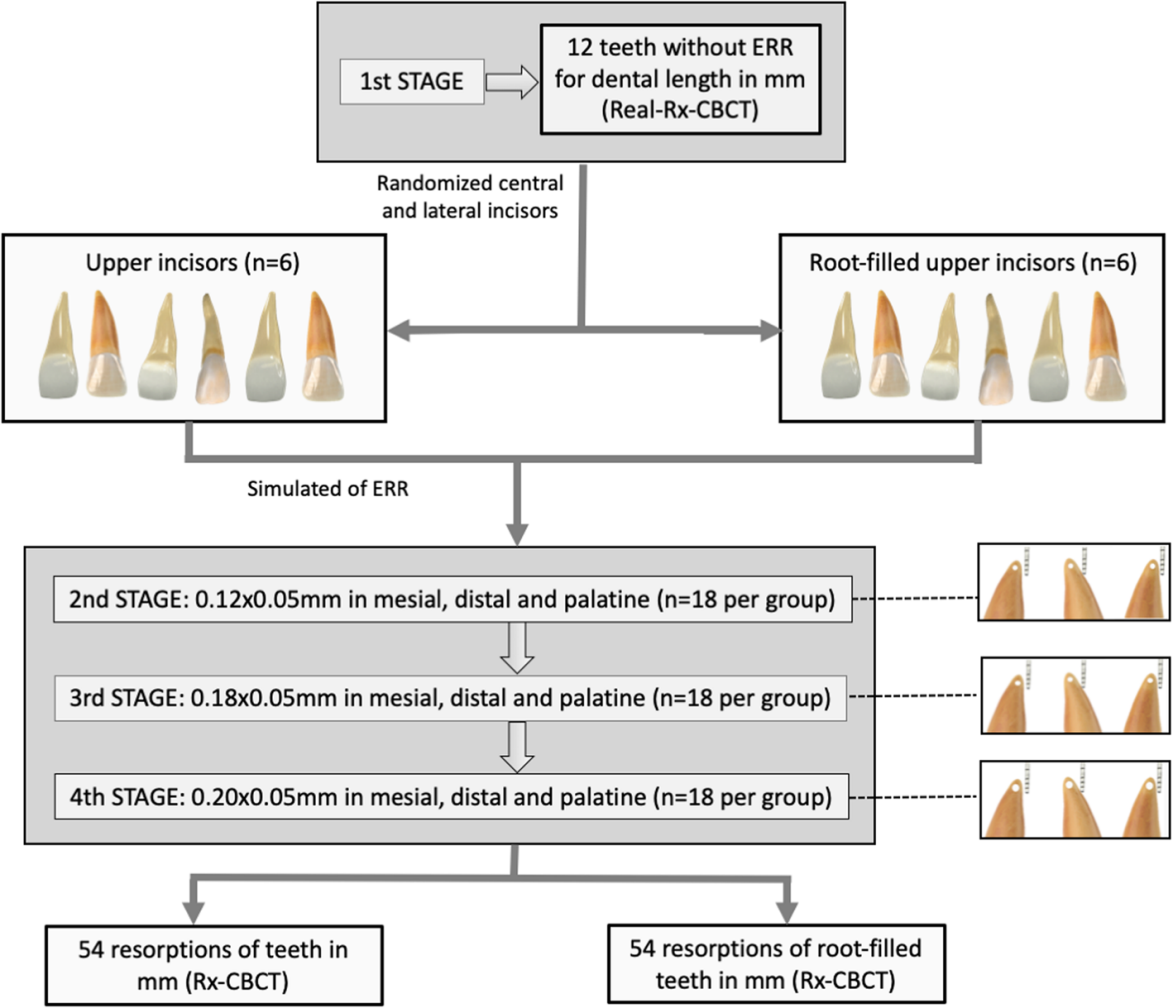
Schematic illustration of the methodology workflow used

Twelve standardized, synthetic replicates of central maxillary and lateral incisors were randomly divided into two groups: (G1) six non-endodontic teeth and (G2) six root canal-treated teeth. To prevent the morphology of the maxillary incisors from influencing the visualization of resorptions, the allocation of tooth type to each group was randomized. After randomization, four central and two lateral incisors were assigned to group 1 and three central and three lateral incisors to group 2. This randomization was applied to determine the root canal morphology of each group. Figure 1 summarizes the workflow of the study.

### Generation of standardized synthetic replicas

Teeth were prepared from a radiopaque synthetic resin *Acry C&B Rx* (Ruthinium Group, RO, Italy) presentation powder and self-healing liquid. Mixing time was 6 min and polymerization time 10 min. The procedure was performed from two high-precision *Hydrorise* silicone molds (Monophase, Zhermack, RO, Italy). The molds were polymerized in a pot under 2 atm. of pressure. Water temperatures ranged between 45^O^C—50^O^C, while the teeth remained inside their respective containers. The synthetic specimens were appropriate and reliable for the purposes of the study. All synthetic replicas were evaluated and measured to quantify and guarantee the accuracy of the six replicas of each central and lateral incisor generated *(Data not shown).*

The root canals of the six tooth specimens in group 2 were then treated by a single operator, using Tagger’s hybrid crown-down technique. Filling procedures were prepared using standardized *GP cones* (Dentsply, Rio de Janeiro, Brazil) with *AH Plus sealer* (Dentsply, Konstanz, Germany). Proper condensation of the maxillary incisors was determined by visualization with digital periapical radiography (DPR) and cone-beam computed tomography (CBCT). Digital measurements (Gocheer, Shenzhen, China) were taken to quantify the precise length of all treated and non-treated tooth specimens from apex to incisal edge.

### Interventions and allocation: *external root resorption simulation*

The replicas were manipulated at the root apex to artificially create root shortening with different tooth lengths. The same operator [21] simulated external root resorption 4 mm from the root apex. Pits were positioned perpendicular to the surface, with high-speed rotation and constant cooling, and the same procedure was repeated at the second, third, and fourth stages. At each stage, artificial ERR lesions were artificially created with three different pit sizes (0.12 mm, 0.18 mm, 0.20 mm) in the mesial, distal, and palatal positions, and 0.05 mm depth [14]. All drill bits had an active length of 0.05 mm and an average grain size of 100 μm. At each stage, *digital periapical radiography* (DPR) and *cone-beam computed tomography* (CBCT) images were taken after sequential creation of the lesions.

### Radiographic exposure and generation of diagnostic images: DPR and CBCT images

Radiographic images were taken after placing each pair of teeth on a frame with a universal radiographic film positioner. The support system was placed parallel to the ground and checked with an *iHandy* spirit level (iHandySoft Inc 2017©, version 1.70.3 for iOS 10.3).

Digital periapical radiographs were obtained with *Timex 70E wall-mounted* (Gnatus, SP, Brazil) X-ray equipment, using 0.32 s. of exposure. The phosphor plates were then read using the *Digora® Optime* system (Soredex, Tuusula, Finland*)* and *Scanora* 5.1.2 *software,* free version for Windows.

CBCT images were obtained using *i-CATTM tomography* (Imaging Sciences International LLC, Hatfield, PA, USA) and free-to-use *iCATVision Q software*, 1.8.1.10 for Windows. Exposure parameters were mAs = 37.07 and 120 KVP, and resolution was 0.20v—26.9 s [22]. The reconstructed volume size was 16 cm diameter x 6 cm depth. In the clinical setting, natural radiation attenuation and scattering is the result of the presence of soft tissue [23]. To simulate this effect, CBCT scans were captured while the block sections were submerged in water in a clear 13 × 9x 5 cm plastic container [23]. All images were obtained by an expert radiologist.

### Radiographic assessment and measurements

**Fig. 2:**
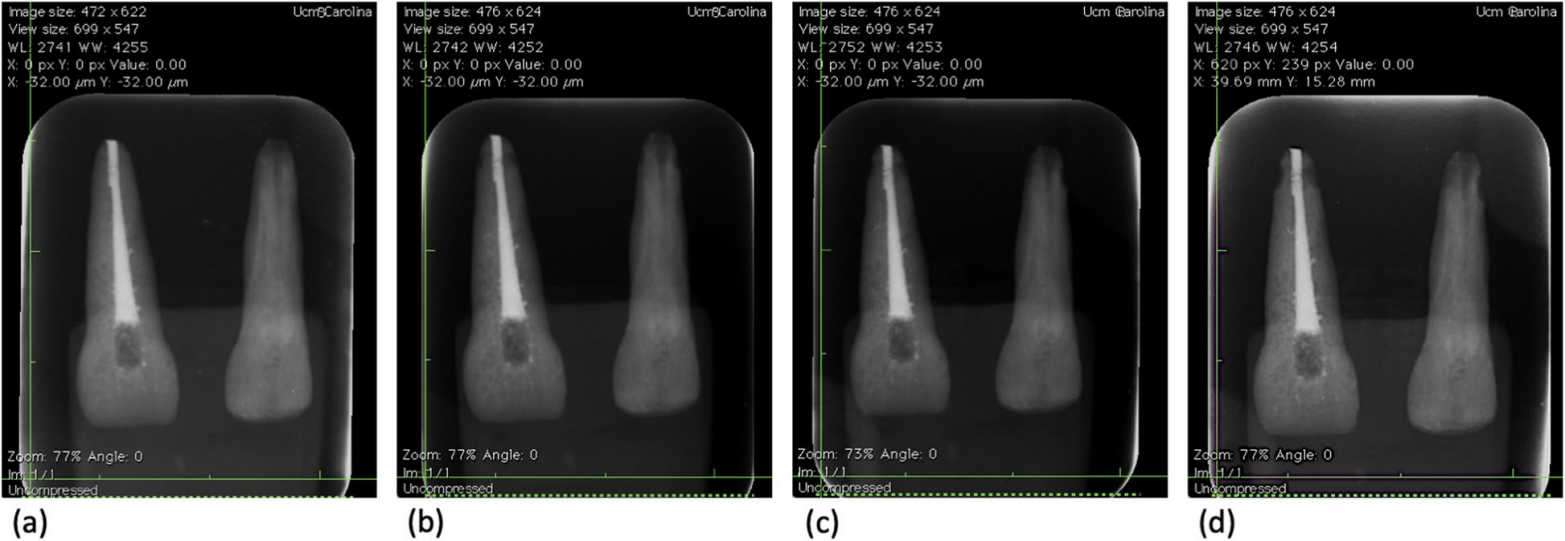
Illustration of endodontic tooth no.1 and non-endodontic tooth no.2 on DPR images. **a** Stage 1, teeth without resorption. **b** Stage 2, teeth with ERR of 0.12 mm. **c** Stage 3, teeth with ERR of 0.18 mm. **d** Stage 4, teeth with ERR of 0.20 mm

**Fig. 3:**
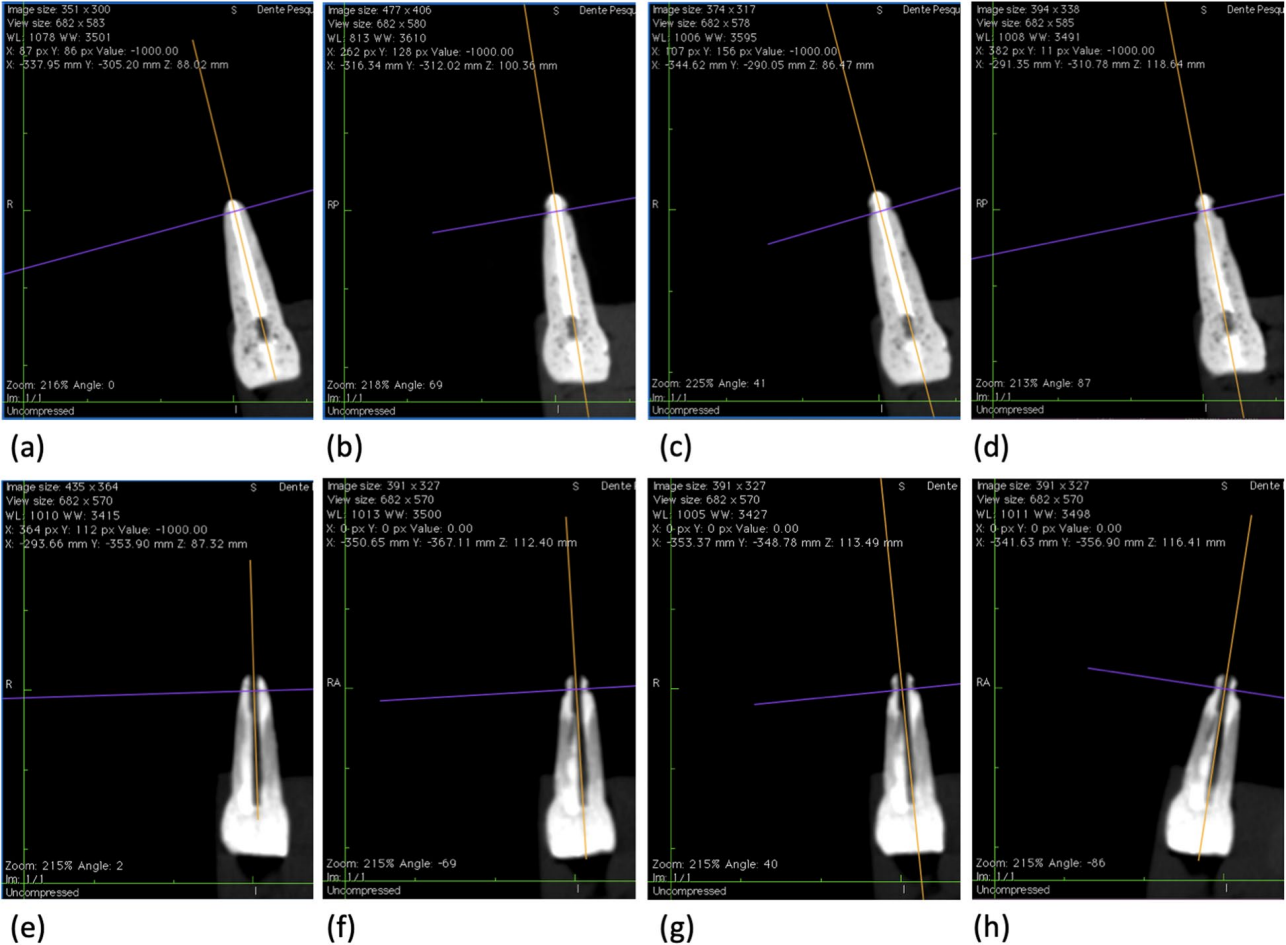
Illustration of endodontic tooth no.1 and non-endodontic tooth no.5 in the coronal plane on CBCT images. **a** Stage 1, tooth no.1 without resorption. **b** Stage 2, tooth no.1 with resorption of 0.12 mm diameter. **c** Stage 3, tooth no.1 with resorption of 0.18 mm diameter. **d** Stage 4, tooth no.1 with resorption pits of 0.20 mm diameter. **e** Stage 1, tooth no.5 without resorption. **f** Stage 2, tooth no.5 with resorption of 0.12 mm diameter. **g** Stage 3, tooth no.5 with resorption of 0.18 mm diameter. **h** Stage 4, no. 5 tooth with resorption of 0.20 mm diameter

**Fig. 4:**
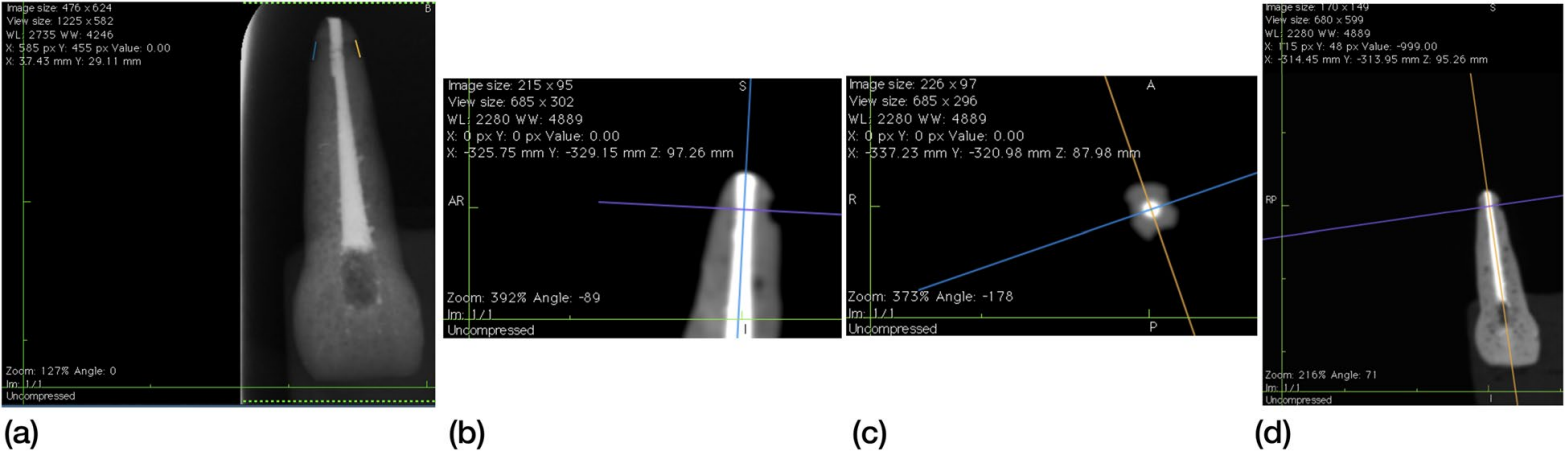
Illustration of measurements on DPR and CBCT images of endodontic tooth no.1. **a** Measurements of mesial and distal ERR on DPR images. **b** Location of guides for measuring the diameter of palatal ERR in the sagittal plane on CBCT images. **c** Axial plane view to help visualize the mesial, distal, and palatal resorption pits on CBCT images (**d**) Guides for measuring diameters of mesial and distal ERR in the coronal plane on CBCT images

The DPR and CBCT images were evaluated by eight previously calibrated and blinded investigators [24–26] using *Horos Software* (GNU, General Public License), version 3.3.6 for Mac (Figs. 2, 3). To minimize bias, observers were blinded to the aims of the study. The observers then adjusted the *Digital Imaging and Communication on Medicine* (DICOM) files according to their needs using the brightness, contrast, and zoom tools. The same dark room was used to analyze the images from all available planes [14]. The longitudinal axis of the maxillary incisors is illustrated in Fig. 4a. In addition, each calibrated evaluator generated a single measurement in the sagittal plane of the ERR on the CBCT images and two measurements in the coronal plane, as illustrated in Fig. 4b-d. The specimens and groups distribution is explained in Supplementary material. Appendix I.

### Statistics

#### Method error

The consistency of the measurements was assessed using the *intraclass correlation coefficient* (ICC), with their respective 95% confidence intervals. To determine intra-examiner agreement, each examiner measured all tooth lengths and external root resorptions twice. Inter-examiner reliability was calculated and the mean of the eight raters’ measurements was selected for further data analysis [21].

#### Comparative, post-hoc analysis and diagnostic method testing

Descriptive statistics were calculated for each group using means and standard deviation. The *Shapiro–Wilk* test was used to determine the goodness-of-fit of the data to a normal distribution. *Repeated measures ANOVA* was calculated. *Univariate analysis of variance* was applied to determine the effects of location and size of ERR pits on perception. The *Kruskal–Wallis* test was applied to determine whether there were significant differences between the four groups according to endodontic type and diagnostic technique [(i) non-endodontic DPR; (ii) endodontic DPR; (iii) non-endodontic CBCT; and (iv) endodontic CBCT]. The *Wilcoxon rank post-hoc* test was performed for all pairwise comparisons. The *Bonferroni* multiple comparisons procedure was also used to evaluate differences in tooth length according to diagnostic method; significance values were adjusted when applying the *Bonferroni correction* in the multiple comparison tests.

Sensitivity, specificity, area under the curve (AUC) and likelihood ratio (LR) were calculated for each group. The data were recorded on *Excel* 16.16.22 spreadsheets (© 2018 Microsoft, Redmond, WA, USA) for Mac. Commercially available *SPSS Statistics* software (© IBM, SPSS Inc, Chicago, IL, USA), version 25 for Mac was used for data analysis. The level of statistical significance was set at (*p* < 0.05).

## Results

### Accuracy of the method

The ICC values for intra- and inter-examiner agreement are shown in Table 1. The results were statistically significant (*p* < 0.001) for all ICC scores. Based on the results obtained, all eight raters’ measurements showed excellent ICC values, and the means of the eight raters’ measurements were selected for further data analysis.

**Table 1:** Intra- and inter-examiner agreement in measures for dental length and diameter of ERR

Examiner	Intra-examiners ICC score^a^						
	Dental length			ERR diameter of Non-endodontic teeth		ERR diameter of Endodontic teeth	
	Real	DPR	CBCT	DPR	CBCT	DPR	CBCT
1	0.859	0.984	0.893	0.896	0.979	0.885	0.907
2	0.927	0.855	0.918	0.963	0.902	0.860	0.886
3	0.958	0.810	0.956	0.909	0.824	0.820	0.897
4	0.996	0.968	0.991	0.861	0.997	0.895	0.983
5	0.871	0.803	0.865	0.944	0.931	0.894	0.944
6	0.942	0.846	0.911	0.995	0.924	0.883	0.995
7	0.821	0.893	0.959	0.897	0.976	0.943	0.863
8	0.899	0.922	0.985	0.998	0.903	0.970	0.924
Mean [± SD]	0.909 ± 0.058	0.885 ± 0.069	0.935 ± 0.045	0.933 ± 0.050	0.929 ± 0.056	0.894 ± 0.047	0.925 ± 0.046
Inter-examiner ICC score^a^	0.899	0.965	1.00	0.989	0.994	0.989	0.966

a: *p*-values (< 0.05) in all data; *ERR* External root resorption, *ICC* Intraclass correlation coefficient, *SD* Standard deviation

### Mean difference in tooth length by CBCT versus digital periapical radiography (DPR)

Table 2 shows the results of the measurements of specimens without resorption lesions, together with the post-hoc adjustments. The data returned statistically significant differences between DPR measurements versus actual lengths (*p* < 0.001), and also versus *CBCT* measurements (*p* < 0.001). It can be concluded that the *DPR* measurements were overestimated compared to the actual ones (Mean diff = 0.765) and also compared to those made by *CBCT* (Mean diff = 0.768). The absolute length measurements obtained with *CBCT* however did not differ from the actual tooth lengths (Mean diff = -0.002; *p* = 0.573).

**Table 2:** Estimated Marginal Means and pairwise comparisons mean difference for dental length

	Mean [± SD] (mm)	CI 95% (mm)	*P* value^a^
Diagnostic Techniques			
Real	24.819 ± 0.346	24.059–25.580	N/A
DPR	25.584 ± 0.319	24.883–26.285	N/A
CBCT	24.817 ± 0.345	24.057–25.577	N/A
Dental length (Differences)			
DPR – Real	0.765 ± 0.116	0.438–1.092	< 0.001
CBCT – Real	-0.002 ± 0.002	-0.008–0.003	0.573
DPR – CBCT	0.768 ± 0.116	0.439–1.096	< 0.001

a: *p*-values with Bonferroni correction, *SD* Standard deviation, *CI* Confidence interval, *N/A* Not applicable

### Evaluation of external root resorption using CBCT versus digital periapical radiography

The results of the evaluation of specimens with ERR after assessing the effect of location and diameter of root resorption lesions are shown in Table 3. More particularly, there were no statistically significant differences between the *DPR* (*p* = 0.728) and *CBCT* (*p* = 0.411) diagnostic techniques, and there were no statistically significant differences between the location or diameter of ERR lesions and their effect on either of the methods analyzed, DPR or CBCT (*p* = 0.527).

**Table 3:** ANOVA test of between the effects of location and diameter size of ERR lesions

Subjects Effects	DPR [*p* value^a^]	CBCT [*p* value^a^]	Difference DPR-CBCT [*p* value^a^]
Resorption Size^b^	0.000	0.000	0.180
Resorption Site^c^	0.037	0.442	0.088
Resorption Size^b^ * Site^c^	0.728	0.411	0.527

a: *p*-values with Bonferroni correction

b: Size: 0.12 mm, 0.18 mm, 0.20 mm

c: Site: mesial, distal and palatine

Comparisons are shown in Table 4. CBCT diagnosis of ERR lesions in specimens without root canal treatment was significantly more accurate than the diagnoses by DPR on both non-endodontic (*p* = 0.044) and endodontic specimens (*p* = 0.037), respectively. However, these differences were not found when considering the diagnosis of ERR on endodontically treated teeth compared to both types of specimen by DPR (*p* > 0.05; *post-hoc* test with *Bonferroni* correction).

**Table 4:** Pairwise comparisons using Wilcoxon rank sum test with continuity correction data

Diagnostic methods	Non-endodontic DPR	Endodontic DPR	Non-endodontic CBCT
Endodontic DPR	1.000^a^	-	-
Non-endodontic CBCT	0.044^a^	0.037^a^	-
Endodontic CBCT	0.097^a^	0.071^a^	1.00^a^

^a^: *p*-values (< 0.05) are multiplied by the number of comparisons with Bonferroni correction

### Quality of diagnostic tests

**Fig. 5:**
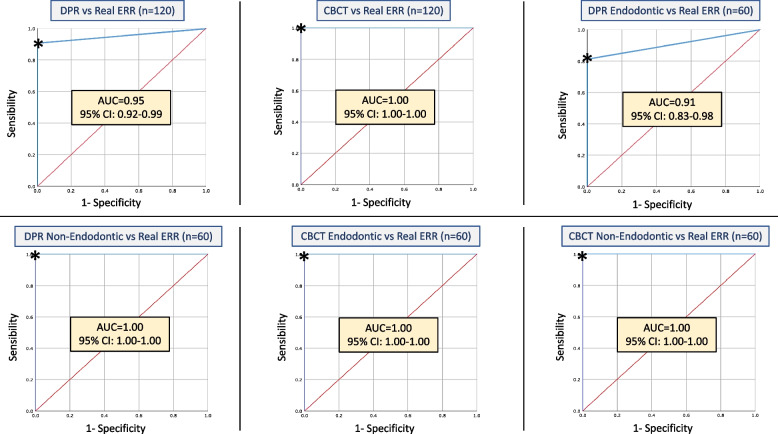
Receiver Operating Characteristic (ROC) curves for the ability to detect ERR in endodontic and non-endodontic teeth using DPR and CBCT

A comparison of the diagnostic tests and their quality is presented in Table 5 and Fig. 5. The sensitivity of DPR for detection of ERR versus the gold standard (true resorption) was 90.7%, with an AUC of 95.4%. However, when the 60 images of endodontic teeth only vs true resorption were selected, sensitivity decreased to 81.5%, with an AUC of 90.7%, versus 100% sensitivity and AUC in teeth without root fillings. With respect to the quality of the diagnostic tests, CBCT obtained an LR- 0.00, and DPR on endodontic teeth obtained an LR- 0.19 (Table 5).

**Table 5:** Comparison and quality of DPR-CBCT diagnostic tests vs Real resorption

Diagnostic methods	N	Sensitivity (%)	Specificity (%)	AUC % (CI %)	Likelihood ratio	
					LR +	LR-
DPR vs Real	120	90.7	100	95.4 (91–99)	999	0.09
CBCT vs Real	120	100	100	100 (100–100)	999	0.00
Non-endodontic DPR vs Real	60	100	100	100 (100–100)	999	0.00
Endodontic DPR vs Real	60	81.5	100	90.7 (83–98)	999	0.19
Non-endodontic CBCT vs Real	60	100	100	100 (100–100)	999	0.00
Endodontic CBCT vs Real	60	100	100	100 (100–100)	999	0.00
Endodontic DPR 0.12 mm vs Real	20	72.2	100	86.1 (72–100)	999	0.28
Endodontic DPR 0.18 mm vs Real	20	75	100	91.7 (80–100)	999	0.17
Endodontic DPR 0.20 mm vs Real	20	88.9	100	94.4 (85–100)	999	0.11

a: *p*-valor (< 0.05) in all data, *AUC* Area under the curve, *CI* Confidence interval 95%

**Fig. 6:**
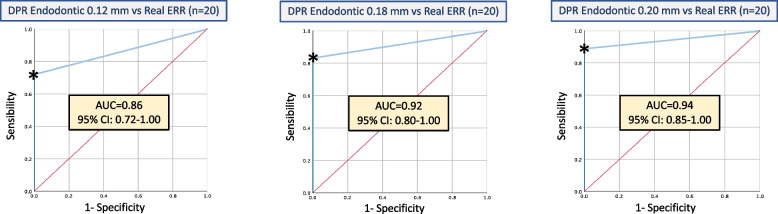
Receiver Operating Characteristic (ROC) curves for the ability to detect incremental ERR lesions (0.12 mm, 0.18 mm, 0.20 mm) in endodontic teeth using DPR

As can be seen in Fig. 6, when DPR was used to assess the graduated ERR lesions, sensitivity increased to 88.9% for the 0.20 lesions, compared to 75% and 72.2% respectively for the 0.18 mm and 0.12 mm lesions, respectively (Table 5).

## Discussion

According to the results obtained, endodontic treatment affects the ability to distinguish ERR. CBCT was proved to be a better and more accurate diagnostic technique than DPR for both types of measurement: estimation of tooth length and assessment of external root resorption lesions. The results showed differential detection of ERR in non-endodontic teeth when analyzed with CBCT compared to teeth with and without root canal treatment analyzed with DPR.

Two main aspects, physical and optical, can affect the perception of ERR when a tooth has received root canal treatment. Among the physical factors, some authors [16] have stated that the differential observation of images depends on the exposure time, X-ray time and radiographic equipment used. The radiation source can also affect radiopacity in the detection and diagnosis of ERR. The density of radiographic noise-generating materials such as fillers, metal posts, implants, and restorations can contribute to radiographic distortion [16]. The high density of gutta-percha interferes with the radiation node more than in non-endodontic teeth. In this study, we compared filled and intact root canals, and ERR was more clearly visible in the non-endodontic teeth. A surface as radiopaque as that of a root-filled tooth makes it very difficult to visually inspect adjacent structures.

Among the optical factors, the quality of the radiographic image can be severely affected by dental angulations and torsions [18]. The location and shape of the ERR should also be taken into account. The compact type of bone in the mandible and the cancellous bone in the upper jaw also potentially influence the diagnosis of ERR. Some authors [17, 18] have stated that lesions confined to the cancellous bone are more difficult to detect early. ERR involves loss of mineral tissue influenced by the action of the osteoclast cells. Some authors have mentioned [27] that changes in density will only be visible on periapical radiographs after considerable resorption. In an in vivo study [17] comparing DPR and CBCT images of 156 endodontically-treated human teeth, the results showed that the zygomatic process of the maxilla can limit the identification of radiographic periapical lesions.

After an in vivo study [18] conducted on 35 endodontically treated teeth, it was concluded that the root resorption lesion had to constitute almost 50% to 60% of bone mineral loss before it could be detected radiographically. One in vitro study [1] investigated resorption in fifteen maxillary anterior teeth with a single root and single canal. The authors argued that 60 to 70% of the mineralized trabecular bone had to be lost before resorption could be detected on a radiograph and that DPR images should be used with caution for the purpose of an ERR diagnosis. With respect to type of radiographic method, some researchers [25, 28] have concluded that CBCT can detect ERR in root-filled teeth more easily and with greater precision than DPR. DPR may be affected to some extent by distortion due to incorrect positioning or angulation during radiographic projection. In the present experiments however, the design and implementation of a digital angle positioner for capturing DPR images ensured that factors of this sort did not alter the sequence of images.

It would have been more desirable to use upper incisor teeth in this study, but these are the ones that are most commonly damaged [26]. However, the radiopacity and radiolucency of the synthetic maxillary incisors matched dental tissue, as was verified by an expert radiologist who compared DICOM images of an ex vivo tooth with a random tooth selected from this research study. This implies that the synthetic teeth in this study were satisfactory. One of the main advantages of using synthetic teeth is that no ethics committee approval is required, which saves time for researchers in this field. To the best of our knowledge, the use of synthetic teeth has not been reported previously. More specifically, this is the first in vitro study to compare CBCT and DPR in the detection of EER on non-endodontic and endodontic teeth using synthetic maxillary incisors.

The methodology used in this research was similar to one proposed by previous authors [26], who simulated external inflammatory resorption on non-endodontic mandibular incisors. In the present study, we included endodontically treated teeth in order to compare them with non-endodontic teeth. This is the first in vitro study to date to compare the detection of ERR on non-endodontic and endodontic teeth using CBCT and DPR. Equally importantly, in the present study, we worked with previously trained and calibrated examiners with expertise in dental radiology and *Horos software* tools. Various authors [25, 29] have argued that expertise is of critical importance in the detection of ERR in both the DPR and CBCT types of projection, DPR in particular.

At the same time, this research study adopted a widely-used experimental model based on root resorption produced by round burs [14, 15]. Three sizes of root resorption were chosen to make comparisons and for detection power, particularly when the smallest drill was used. Based on the results, CBCT was a better diagnostic technique than DPR. Our findings are corroborated by a number of medical studies [23, 26, 28–30], although the differences we observed between detection of ERR on non-endodontic and endodontic teeth are, to the best of our knowledge, a novelty in the field. In the present study, a DPR diagnosis was observed to be 18.5% less sensitive on endodontically-treated versus non-endodontic teeth. The decrease in sensitivity was even more marked on the smallest ERR lesions, with 27.8 and 25% less sensitivity, respectively.

We speculate that these results are due to the radiopacity of endodontic teeth, which results in greater root brightness than in non-endodontic teeth. Apart from that, DPR was studied using two-dimensional images and, for all the reasons mentioned above, it is particularly difficult to detect ERR on DPR images. In all 2D image experiments, palatal resorptions were only detected on non-endodontic and endodontic teeth with the largest drill bit. Overall, detection of ERR in endodontically-treated teeth may be underdiagnosed and more careful inspection is required than when screening for ERR in vital teeth.

## Conclusions

The results of this study highlight that both CBCT and DPR are good diagnostic methods for ERR. Nevertheless, root-filling material modifies the diagnostic capacity of ERR and makes the use of DPR less accurate in the detection and diagnosis of ERR than in non-endodontic teeth.

## Supplementary Information

**Additional file 1**: **Supplementary material Appendix I.** Specimens and groups distribution.

## Data Availability

The datasets used and/or analysed during the current study available from the corresponding author on reasonable request.
