# Supplementary material for: Does endodontics influence radiological detection of external root resorption? an in vitro study
Source: BMC Oral Health. 2023 Apr 17;23:221. doi: 10.1186/s12903-023-02871-w (PMC10108466; doi:10.1186/s12903-023-02871-w)
Supplement: Supplementary file 1 — Additional file 1: Supplementary material Appendix I. Specimens and groups distribution. [file 12903_2023_2871_MOESM1_ESM.docx]

**Supplementary material. Appendix I. Specimens and groups distribution.**

To sum up, the measurements were assessed in six sessions: (i) 12 teeth for dental length measures through the *Digital Vernier caliper* (Gocheer, Shenzhen, China); (ii) 24 Digital Periapical Radiographs through the *Horos Software*; (iii) 96 CBCT scans through the Software mentioned; (iv) repeat all dental length measures; (v) repeat all radiographs measures; and (vi) repeat all CBCT scans measures. Consequently, the last three sessions were carried out after three weeks to assess the intra-examiner agreement.

Moreover, regarding the quality of the diagnostic test, were assessed a total of 120 images of DPR, CBCT and gold standard (real ERR). Then, these images were dividing into six groups: (i) 60 DPR of non-endodontic teeth; (ii) 60 DPR non-endodontic teeth; (iii) 60 CBCT endodontic teeth; (iv) 60 CBCT non-endodontic teeth versus (v) 60 gold standard endodontic teeth; and (vi) 60 gold standard non-endodontic teeth images.

Finally, giving a thorough look, it divided into six groups more according to the lesions of ERR in DPR images and compared with the gold standard. The additional groups were: (vii) 20 DPR endodontic teeth with 0.12mm; (viii) 20 DPR non-endodontic teeth with 0.12mm; (ix) 20 DPR endodontic teeth with 0.18mm; (x) 20 DPR non-endodontic teeth with 0.18mm; (xi) 20 DPR endodontic teeth with 0.20mm; (xii) 20 DPR non-endodontic teeth with 0.20mm.
